# Cost effectiveness of group follow-up after structured education for type 1 diabetes: a cluster randomised controlled trial

**DOI:** 10.1186/1745-6215-15-227

**Published:** 2014-06-14

**Authors:** Paddy Gillespie, Eamon O'Shea, Mary Clare O’Hara, Sean F Dinneen

**Affiliations:** 1 School of Business and Economics, J.E. Cairnes Building, NUI Galway, Galway, Ireland; 2 School of Medicine, NUI Galway, Galway, Ireland

**Keywords:** Type 1 diabetes, Structured education, Follow-up, Cost effectiveness

## Abstract

**Background:**

This study examines the cost effectiveness of group follow-up after participation in the Dose Adjustment for Normal Eating (DAFNE) structured education programme for type 1 diabetes.

**Methods:**

Economic evaluation conducted alongside a cluster randomised controlled trial involving 437 adults with type 1 diabetes in Ireland. Group follow-up involved two group education ‘booster’ sessions post-DAFNE. Individual follow-up involved two standard one-to-one hospital clinic visits. Incremental costs, quality-adjusted life years (QALYs) gained and cost effectiveness were estimated at 18 months. Uncertainty was explored using sensitivity analysis and by estimating cost effectiveness acceptability curves.

**Results:**

Group follow-up was associated with a mean reduction in QALYs gained of 0.04 per patient (*P* value, 0.052; 95% CI, −0.08 to 0.01, intra-class correlation (ICC), 0.033) and a mean reduction in total healthcare costs of €772 (*P* value, 0.020; 95% CI, −1,415 to −128: ICC, 0.016) per patient. At alternative threshold values of €5,000, €15,000, €25,000, €35,000, and €45,000, the probability of group follow-up being cost effective was estimated to be 1.000, 0.762, 0.204, 0.078, and 0.033 respectively.

**Conclusions:**

The results do not support implementation of group follow-up as the sole means of follow-up post-DAFNE. Given the reported cost savings, future studies should explore the cost effectiveness of alternative models of group care for diabetes.

**Trial registration:**

Current Controlled Trials ISRCTN79759174 (assigned: 9 February 2007).

## Background

The Dose Adjustment for Normal Eating (DAFNE) programme, which comprises of group-based structured education sessions, has become an established strategy for enhancing self-management in individuals with type 1 diabetes [1]. The existing evidence base suggests that the DAFNE programme is both cost saving and more effective when compared with conventional treatment [2,3]. This notwithstanding, questions remain as to how follow-up care should be provided post-DAFNE in order to maintain its benefits over the longer term. The Irish DAFNE study sought to address this issue directly by conducting a cluster randomised controlled trial (RCT) to explore the clinical and cost effectiveness of group follow-up versus individual follow-up after participation in the DAFNE programme [4]. Notably, the study did not evaluate DAFNE directly; rather it evaluated two approaches to follow-up care post-DAFNE. This paper reports the findings for the cost effectiveness analysis.

Full details of the trial methods are published elsewhere [5]. In brief, a cluster RCT recruited six hospital clinics and 437 patients with a diagnosis of type 1 diabetes (Current Controlled Trials ISRCTN79759174). Three clinics were randomised to usual care, consisting of individual follow-up, where participants (n = 221) were invited to attend outpatient clinics at 6 and 12 months post-DAFNE for one-to-one visits with a doctor, nurse and/or dietitian. Three clinics were randomised to group follow-up, in which participants (n = 216) returned in their original DAFNE group and received “booster” education sessions from programme educators at 6 and 12 months post-DAFNE. A structured curriculum was developed to facilitate the group follow-up sessions. This comprised of a set of learning objectives across a range of topics that were “offered” to participants on the basis of their perceived need. Goal setting and action planning was emphasised as was the reviewing of patients’ blood sugar records (Table 1).

**Table 1:** The Irish DAFNE Study: Describing the Intervention

DAFNE course	The DAFNE course is delivered over 5 consecutive days to groups of up to 8 individuals who are using a basal/bolus insulin regimen to manage their diabetes. It involves 38 hours of structured education covering all aspects of diabetes self-management with an emphasis on carbohydrate estimation and matching of quick-acting insulin to food.
	The course is delivered by a DAFNE-trained diabetes nurse, dietitian and doctor, who are regularly peer reviewed to ensure that the education is consistently delivered according to the curriculum.
	All groups are invited back to a 3 hour review session at 6 weeks post-DAFNE to consolidate skills learned and to review targets and goals.
Group follow up	Intervention arm participants met at 6 and 12 months post-DAFNE in the original group to which they were assigned. Group follow-up sessions lasted approximately 3 hours.
	Sessions were facilitated by trained educators using a structured curriculum, which included topics such as principles of insulin dose adjustment, carbohydrate estimation and managing hypoglycaemia. Groups identified their own priorities for discussion while the educator used the curriculum to guide the session.
	Participants were encouraged to reflect on progress and difficulties with their original self-management goals and to produce an updated action plan.

Details on the baseline characteristics of the participating patients in each arm are presented in Table 2. Eighteen patients in the intervention group and 23 patients in the control group were lost to follow-up, leaving 398 (91%) patients in the analysis. The primary outcome in the clinical effectiveness analysis was change in glycated haemoglobin (HbA_1c_) from baseline to follow-up. At 18 months, the trial showed no clinically or statistically significant difference in HbA_1c_ between groups (effect, 0.14; *P* value, 0.470; 95% CI, −0.33 to 0.61; see Additional file 1: Table S1) [4]. The study concludes that group follow-up was as effective as a one-to-one individual clinic follow-up after structured education for type 1 diabetes.

**Table 2:** Baseline characteristics of study participants by treatment arm

**Characteristic**	**Control**	**Intervention**
	**Individual follow-up**	**Group follow-up**
N	221 (51)	216 (49)
Age (years)	41.5 (11.4)	40.1 (12.0)
Gender		
Male	127 (57.5%)	108 (50%)
Female	94 (42.5%)	108 (50%)
Marital status		
Married/cohabiting	106 (59.6%)	130 (65%)
Other	72 (40.4%)	70 (35%)
Education status		
Completed 3rd level	96 (55.2%)	82 (41.6%)
Other	78 (44.8%)	115 (58.4%)
Employment status		
Employed	136 (76.4%)	141 (70.1%)
Retired	6 (3.4%)	5 (2.5%)
Other	36 (20.2%)	55 (27.5%)
Years since diagnosis	16.3 (11.2)	15.5 (10.4)
Baseline BMI (kg/m^2^)	26.3 (4.3)	25.8 (4.0)
Baseline HbA_1c_ (%)	8.2 (1.3)	8.4 (1.4)
Baseline HbA_1c_ (mmol/mol)	66 (9.1)	68 (9.8)
Smoking status		
Smoker	37 (20.6%)	42 (20.8%)
Non-smoker	143 (79.4%)	160 (79.2%)
Comorbidity status	75 (43%)	64 (33%)
Selected comorbidity status		
Heart disease	4 (2%)	8 (4%)
High blood pressure	32 (18%)	10 (5%)
Chest/lung disease	3 (2%)	9 (5%)

Values are shown as mean (SD) or n (%) as appropriate. BMI, body mass index; HbA_1c_, glycated haemoglobin. Data from Dinneen and colleagues [4].

In addition to clinical effectiveness, any decision regarding the adoption of a healthcare intervention in clinical practice will depend upon its expected cost effectiveness [6]. The technique of economic evaluation explores cost effectiveness by relating the mean difference in cost between alternative treatment options to their mean difference in effectiveness, and by quantifying the uncertainty surrounding these incremental point estimates. This paper reports the cost effectiveness results from an economic evaluation conducted alongside the cluster RCT to compare group follow-up versus individual follow-up after participation in the DAFNE programme for type 1 diabetes.

## Methods

### Overview

The economic evaluation was conducted following the guidelines for health technology assessment for Ireland [7]. It consisted of a trial-based analysis with a time horizon of 18 months - the trial follow-up period. Ethical approval for the study was provided by the local committees at the participating study centres (Galway University Hospitals; NUI Galway; Health Service Executive Dublin Mid-Leinster; Beaumont Hospital, Dublin; St Vincent's Healthcare Ethics and Medical Research Committee, Dublin; Office for Research Ethics Committee, Northern Ireland). Informed consent was obtained from each participant. The perspective of the healthcare provider was adopted with respect to costing and health outcomes were expressed in terms of quality-adjusted life years (QALYs). Data on resource use and health status were collected via structured patient questionnaires at baseline and at three follow-up points: 6 months, 12 months and 18 months. Given the length of follow-up, neither costs nor outcomes were discounted.

The statistical analysis was conducted on an intention-to-treat basis, and in accordance with current guidelines for cluster RCTs [8-13]; that is, we adopt multilevel statistical techniques which recognise both the clustering and correlation in the cost and effect data. Descriptive statistics, in the form of means, standard deviations and intra-correlation (ICC) coefficients, were estimated for the variables of interest. The incremental analysis was undertaken using multivariate multilevel regression techniques. Separate regression models were estimated for costs and health outcomes, both of which were estimated controlling for treatment arm, clustering, and a range of other covariates and factors [11]. Uncertainty in the analysis was addressed by estimating 95% CIs and cost effectiveness acceptability curves (CEACs), which link the probability of treatment being cost effective to a range of potential threshold values (λ) that a health system may be willing to pay per additional QALY gained [14]. The CEACs were estimated using a two-stage bootstrapping technique [13], which jointly accounts for the clustering and correlation in the cost and effect data. In addition, a series of sensitivity analysis was conducted. All analysis was undertaken in the Stata 13 statistical software package (StataCorp LP, USA).

### Cost analysis

Two cost components were included in the analysis, all of which were expressed in Euros (€) at 2009 prices. The first related to the cost of implementing the group follow-up intervention in clinical practice. This included a range of resources such as educator and administrator time input, educational materials and consumables, post, packaging, telephone and travel expenses. This data was recorded prospectively by the study research team. The total cost of implementing the group follow-up intervention was €39,933, giving a mean cost per participant estimate of €185. This cost was allocated to all patients in the group follow-up arm.

Second, costs relating to the use of primary and secondary healthcare services over the course of the trial were estimated for patients in both treatment arms. This included the costs of general practitioner, dietitian, and chiropodist consultations, diabetes day care centre, outpatient, and accident and emergency visits, hospital admissions, medications including insulin, lipid lowering, antiplatelet, and antihypertensive therapies, and blood glucose self-monitoring tests. Resource use was captured via structured questionnaires completed by patients at baseline and the three follow-up data collection points: 6, 12 and 18 months. A vector of unit costs was applied to calculate the cost associated with each resource activity at each time point. Notably, the baseline time point included the period during which the DAFNE programme was delivered. Unit cost estimates for each activity were based on national data sources and, where necessary, were transformed to Euros (€) at 2009 prices using appropriate indices [15] (see Table 3). In sensitivity analysis, the effects of deflating the unit cost inputs by 10% and 50% were examined.

**Table 3:** Categories of resource use and unit cost estimates in 2009 (€) prices

**Healthcare resource item**	**Activity**	**Unit cost (€)**	**Source**
GP clinic	Per visit	50	ORC
Hospital admission	Per inpatient day	832	DOHC
Outpatient clinic	Per visit	169	DOHC
Accident and emergency clinic	Per visit	289	DOHC
Diabetes nurse	Per consultation	27	HSE
Dietitian	Per consultation	24	HSE
Chiropodist	Per consultation	24	HSE
Diabetes centre clinic	Per visit	169	DOHC
Quick-acting insulin	Per IU	0.02	MIMS
Background insulin	Per IU	0.03	MIMS
Blood glucose monitoring	Per test	0.39	NICE
Lipid lowering therapy	Per day	1.33	MIMS
Antiplatelet therapy	Per day	0.56	MIMS
Antihypertensive therapy	Per day	0.74	MIMS

CSO, Central Statistics Office, Dublin, Ireland; DOF, Department of Finance, Dublin, Ireland; DOHC, Casemix Unit, Department of Health and Children, Dublin, Ireland; GP, general practitioner; HSE, Salary Scales, Health Service Executive, Dublin, Ireland; IU, insulin unit; MIMS, Monthly Index of Medical Specialties Ireland, Dublin, Ireland; NICE, National Institute of Clinical Excellence, London, United Kingdom; ORC, Office of the Revenue Commissioner, Dublin, Ireland.

For the purposes of the incremental analysis, a total healthcare cost at 18 months follow-up variable was constructed. The cost per individual resource activity at 18 months follow-up was calculated by aggregating individual resource costs across the three follow-up periods: 0 to 6 months, 6 to 12 months, and 12 to 18 months. The individual resource costs were then summed to compute the total healthcare cost variable. To facilitate this process, multiple imputation [12] was undertaken using the *MI* package in Stata 13 to estimate missing values for individual resource use cost data at each time point. The imputation models for each variable included age, gender, length of illness, treatment arm, and hospital clinic, were estimated using predictive mean matching, and were based on *M =* 5 imputed data sets.

Estimation of incremental total healthcare cost was undertaken using a linear mixed effects regression model. The model was estimated controlling for treatment arm, baseline cost, age, gender, length of illness, HBA_1c_, body mass index, heart disease status, high blood pressure status, chest or lung disease status, smoking status, medical card status, marital status, education status, employment status, and clustering. The analysis was undertaken using the *MI estimate mixed* and *MI predict* commands in Stata 13, which estimate the regression model on each of the imputed data sets and apply Rubin’s rules to generate the coefficients of interest [16]. To account for the hierarchical and distributional nature of the total cost variable, the regression models were estimated with an exchangeable correlation structure and with robust standards.

The adoption of two alternative model specifications for the cost analysis was examined in sensitivity analysis. First, a parsimonious model, controlling for treatment arm, baseline cost, and clustering was estimated. Second, a generalised linear model with cluster standard errors, assuming a Gamma variance function and identity link function, was estimated on the full set of covariates and factors. Notably, multilevel models based on the Gamma distribution may fit cost data better than Gaussian based approaches [17]. We report findings from both approaches for comparison.

### Effectiveness analysis

Health outcomes in the analysis were expressed in terms of QALYs gained, calculated using the EuroQol EQ5D 3 L instrument [18,19], a standardised tool designed to describe and value health status. The EQ5D 3 L consists of five dimensions: mobility, self-care, usual activities, pain or discomfort and anxiety or depression; and each dimension has three levels of severity: no problems, moderate problems or extreme problems. A scoring algorithm is applied to transform EQ5D responses into a single health state index score, which typically range from 0 (equivalent to death) to 1 (equivalent to good health), although a small number of health states are valued as worse than death. The scoring algorithm is based on values elicited via a time trade-off approach for the UK population [20,21]. Quality-adjusted life expectancy over a period of time is calculated by weighting each component of the time period by its relevant health state index score, using the area under the curve method [22].

For the purposes of the incremental analysis, a 'QALYs gained at 18 months' variable was constructed using the EQ5D scores for each participant at baseline, 6, 12, and 18 months using the area under the curve method. Once again, to facilitate this process, multiple imputation was undertaken using the *MI* package in Stata 13 to estimate missing values for EQ5D scores at each time point. The imputation models for each variable included age, gender, length of illness, treatment arm, and hospital clinic, were estimated using predictive mean matching, and were based on *M =* 5 imputed data sets.

Estimation of incremental QALYs gained was undertaken using a linear mixed effects model. The model was estimated controlling for treatment arm, baseline EQ5D score, age, gender, length of illness, HBA_1c_, body mass index, heart disease status, high blood pressure status, chest or lung disease status, smoking status, medical card status, marital status, education status, employment status, and clustering. As for the cost analysis, the analysis was undertaken using the *MI estimate mixed* and *MI predict* commands in Stata 13.

In sensitivity analysis, a parsimonious model, controlling for treatment arm, baseline EQ5D score, and clustering, was estimated.

### Cost effectiveness analysis

In economic evaluation, a treatment can be defined as more cost effective than a comparator if one of the following conditions apply: (a) it is less costly and more effective; (b) it is more costly and more effective, but its additional cost per additional unit of effect, known as the incremental cost effectiveness ratio, is considered worth paying by decision makers; or (c) it is less costly and less effective, but the additional cost per additional unit of effect of its comparator is not considered worth paying by decision makers [6]. To identify which scenario applies in any case, the incremental estimates for the differences in mean cost and effectiveness between the treatment alternatives must be examined.

To undertake cost effectiveness analysis alongside cluster RCTs, techniques which recognise the clustering and correlation in the cost and effect data must be adopted [9]. In this case, separate multilevel regression models, controlling for treatment arm, clustering, and a range of other covariates and factors [11], were used to estimate the incremental mean costs and QALYs for group follow-up relative to individual follow-up. The uncertainty surrounding these point estimates was examined using a two-stage non-parametric bootstrapping technique estimated using the *TSB* command in Stata 13 [13]. This method explicitly accounts for the correlation and clustering in the hierarchical cost and effect data. The results are presented using CEACs, which report cost effectiveness probabilities for a range of potential threshold values. In doing so, the CEACs incorporate both the sampling uncertainty around the mean cost effectiveness estimates and the uncertainty around the cost effectiveness threshold value, [14], which is unknown for Ireland [23].

## Results

Raw data estimates for resource use and EQ5D scores at baseline and each follow-up time point are presented in Table 4. The equivalent unadjusted estimates for resource costs and QALYs gained are summarised in Table 5. Details on missing data at each time point are presented in Additional file 1: Table S2.

**Table 4:** Resource use and EQ5D estimates at baseline and follow-up by treatment arm

**Variable/time point**	**Baseline: 12 months**		**Follow-up 1: 0 to 6 months**		**Follow-up 2: 6 to 12 months**		**Follow-up 3: 12 to 18 months**	
	**Individual follow-up**	**Group follow-up**	**Individual follow-up**	**Group follow-up**	**Individual follow-up**	**Group follow-up**	**Individual follow-up**	**Group follow-up**
**Resource item**								
GP visits: diabetes	0.50 (1.35)	0.37 (0.93)	0.25 (0.75)	0.17 (0.61)	0.28 (0.72)	0.15 (0.46)	0.25 (0.84)	0.28 (0.91)
GP visits: other	1.35 (1.95)	1.16 (1.72)	1.45 (2.44)	1.12 (1.43)	1.25 (1.61)	1.11 (1.37)	1.19 (2.06)	1.15 (1.42)
Diabetes nurse visits	1.09 (1.03)	0.90 (1.05)	0.72 (1.21)	0.58 (0.76)	0.57 (1.10)	0.43 (0.74)	0.45 (0.70)	0.46 (0.81)
Diabetes nurse calls	0.78 (1.78)	0.61 (1.78)	0.75 (1.42)	0.56 (1.06)	0.51 (1.26)	0.49 (1.06)	0.55 (1.22)	0.44 (1.36)
Dietitian visits	0.39 (0.59)	0.34 (0.74)	0.30 (0.60)	0.28 (0.53)	0.29 (0.67)	0.20 (0.44)	0.16 (0.38)	0.10(0.35)
Dietitian calls	0.05 (0.21)	0.04 (0.21)	0.28 (1.09)	0.10 (0.37)	0.12 (0.46)	0.10 (0.49)	0.05 (0.25)	0.06 (0.55)
Outpatient visits: diabetes	0.84 (0.76)	0.82 (0.65)	0.50 (0.59)	0.36 (0.58)	0.50 (0.60)	0.30 (0.53)	0.49 (0.61)	0.41 (0.62)
Outpatient visits: other	0.29 (0.72)	0.39 (1.55)	0.45 (1.67)	0.33 (0.88)	0.49 (1.29)	0.32 (0.80)	0.43 (0.94)	0.49 (1.17)
Inpatient days: diabetes	0.27 (1.44)	0.17 (0.86)	0.16 (0.95)	0.02 (0.19)	0.04 (0.30)	0.03 (0.16)	0.06 (0.44)	0.11 (0.64)
Inpatient days: other	0.24 (1.03)	0.39 (2.30)	0.29 (1.48)	0.10 (0.42)	0.46 (2.16)	0.22 (1.03)	0.19 (1.06)	0.15 (0.71)
A&E visits: diabetes	0.08 (0.36)	0.06 (0.26)	0.04 (0.18)	0.02 (0.19)	0.01 (0.08)	0.04 (0.20)	0.03 (0.16)	0.03 (0.16)
A&E visits: other	0.07 (0.27)	0.10 (0.36)	0.07 (0.28)	0.14 (0.48)	0.09 (0.33)	0.11 (0.42)	0.06 (0.24)	0.10 (0.35)
Chiropodist visits	0.27 (0.56)	0.25 (0.61)	0.32 (0.62)	0.20 (0.42)	0.31 (0.62)	0.23 (0.56)	0.32 (0.69)	0.23 (0.57)
Diabetes centre visits	1.35 (1.10)	1.25 (1.15)	1.02 (1.33)	0.80 (0.02)	0.92 (1.20)	0.70 (0.90)	0.79 (1.07)	0.72 (0.92)
Quick-acting insulin (IUs)	30.53 (15.15)	27.88 (14.56)	25.39 (13.73)	24.09 (13.57)	26.84 (12.82)	24.09 (15.12)	25.82 (13.14)	25.78 (13.69)
Background insulin (IUs)	25.68 (11.61)	24.35 (14.17)	21.11 (8.94)	19.02 (9.77)	21.44 (9.76)	19.22 (9.46)	21.80 (10.58)	20.39 (11.53)
Blood glucose tests	3.79 (2.38)	3.72 (1.99)	4.46 (1.91)	4.07 (1.43)	4.53 (1.96)	4.37 (1.93)	4.60 (2.04)	4.22 (2.04)
Lipid lowering therapy	67 (30%)	69 (32%)	46 (32%)	53 (36%)	49 (34%)	55 (34%)	48 (33%)	50 (37%)
Antiplatelet therapy	53 (24%)	70 (32%)	43 (30%)	51 (35%)	43 (30%)	54 (33%)	40 (27%)	45 (33%)
Antihypertensive therapy	57 (26%)	76 (35%)	28 (20%)	51 (35%)	32 (22%)	51 (35%)	30 (21%)	49 (36%)
**Health outcome**								
EQ5D score	0.88 (0.20)	0.87 (0.18)	0.91 (0.15)	0.88 (0.16)	0.92 (0.14)	0.86 (0.20)	0.90 (0.16)	0.88 (0.17)

Values are shown as mean (SD) or n (%) as appropriate. A&E, accident and emergency; EQ5D, EuroQol five dimensions; GP, general practitioner; IU, insulin unit.

**Table 5:** Cost and QALY estimates at baseline and follow-up by treatment arm

**Variable/time point**	**Baseline: 12 months**		**Follow-up 1: 0 to 6 months**		**Follow-up 2: 6 to 12 months**		**Follow-up 3: 12 to 18 months**	
	**Individual follow-up**	**Group follow-up**	**Individual follow-up**	**Group follow-up**	**Individual follow-up**	**Group follow-up**	**Individual follow-up**	**Group follow-up**
**Healthcare resources**								
GP visits: diabetes	25 (68)	19 (46)	12 (38)	9 (30)	14 (36)	7 (23)	13 (42)	14 (45)
GP visits: other	68 (98)	58 (86)	73 (122)	56 (72)	62 (81)	56 (69)	59 (103)	58 (71)
Diabetes nurse visits	29 (28)	24 (28)	20 (33)	16 (21)	16 (30)	12 (20)	12 (19)	12 (22)
Diabetes nurse calls	21 (48)	17 (48)	20 (38)	15 (29)	14 (34)	13 (29)	15 (33)	12 (37)
Dietitian visits	9 (14)	8 (18)	7 (15)	7 (13)	7 (16)	5 (11)	4 (9)	3 (8)
Dietitian calls	1 (5)	1 (5)	7 (26)	2 (8)	3 (11)	2 (12)	1 (6)	2 (13)
Outpatient visits: diabetes	143 (128)	139 (110)	85 (99)	60 (98)	85 (101)	50 (89)	83 (103)	69 (104)
Outpatient visits: other	49 (122)	66 (262)	76 (282)	55 (149)	83 (218)	54 (134)	72 (158)	83 (198)
Inpatient days: diabetes	228 (1199)	139 (716)	131 (792)	19 (160)	33 (251)	21 (129)	50 (362)	90 (533)
Inpatient days: other	195 (860)	324 (1913)	242 (1230)	80 (350)	383 (1799)	184 (853)	161 (882)	124 (593)
A & E visits: diabetes	23 (104)	17 (74)	10 (53)	6 (56)	2 (23)	12 (58)	8 (47)	8 (47)
A & E visits: other	20 (79)	30 (105)	20 (80)	41 (138)	26 (96)	31 (122)	17 (69)	29 (100)
Chiropodist visits	7 (13)	6 (15)	8 (15)	5 (10)	8 (15)	6 (13)	8 (17)	6 (14)
Diabetes centre visits	229 (187)	211 (194**)**	172 (225)	136 (156)	156 (203)	118 (153)	133 (180)	122 (156)
Quick-acting insulin	111 (55)	102 (53)	93 (50)	88 (50)	98 (47)	88 (55)	94 (48)	94 (50)
Background insulin	94 (42)	89 (52)	77 (33)	69 (36)	78 (36)	70 (35)	80 (39)	74 (42)
Blood glucose tests	270 (169)	265 (141)	317 (136)	290 (102)	323 (139)	311 (137)	327 (145)	301 (145)
Lipid lowering therapy	76 (113)	79 (114)	78 (114)	87 (117)	82 (115)	89 (117)	80 (115)	89 (117)
Antiplatelet therapy	26 (44)	34 (48)	31 (47)	36 (49)	30 (47)	36 (49)	28 (47)	33 (48)
Antihypertensive therapy	36 (60)	49 (65)	26 (54)	49 (65)	30 (56)	47 (64)	28 (55)	49 (65)
								
**Total cost**								
Total healthcare cost	1,597 (1,549)	1,643 (2,416)	1,413 (1,347)	1,189 (840)	1,343 (1588)	1,246 (1021)	1,274 (1181)	1,283 (1105)
**Health outcome**								
QALYs gained	0.44 (0.09)	0.43 (0.09)	0.45 (0.08)	0.44 (0.07)	0.46 (0.05)	0.43 (0.07)	0.46 (0.06)	0.44 (0.08)

Values are shown as mean (SD) or n (%) as appropriate. A&E, accident and emergency; GP, general practitioner; QALY, quality-adjusted life year.

The results from the incremental cost effectiveness analysis are presented in Table 6. These indicate that group follow-up was, on average, less costly and less effective than individual follow-up. With respect to total healthcare costs at 18 months, the mean cost per patient estimates were €4,337 for the individual follow-up arm and €3,551 for the group follow-up arm. The results from the multilevel regression analysis indicate that group follow-up was associated with a reduction in mean cost of €772 (*P* value, 0.020; 95% CI, −1,415 to −128; ICC, 0.016) per patient. In terms of QALYs gained at 18 months, the mean estimates were 1.35 for individual follow-up and 1.31 for group follow-up. The multilevel regression results indicate that group follow-up was associated with a reduction in mean QALYs of 0.04 (*P* value, 0.052; 95% CI, −0.08 to 0.01; ICC, 0.033) per patient.

**Table 6:** Incremental cost effectiveness analysis results

**Analysis**	**Intervention**	**Control**	**ICC**
	**Group follow-up N = 216**	**Individual follow-up N = 221**	
**Cost analysis**			
**Total healthcare cost (€)**			
Mean (SD)	3,551 (566)	4,337 (551)	0.016
Incremental analysis (difference in means; intervention versus control)	−772 (95% CI, −1,415 to −128; *P* = 0.020)		
**Effectiveness analysis**			
QALYs gained			
Mean (SD)	1.31 (0.12)	1.35 (0.12)	0.033
Incremental analysis (difference in means; intervention versus control)			
	−0.04 (95% CI, −0.08 to 0.01; *P* = 0.052)		
	**Cost effectiveness analysis (probability that treatment is cost effective at λ**		
**Threshold value (λ)**			
λ = €0	1.000	0.000	
λ = €5,000	1.000	0.000	
λ = €10,000	0.996	0.004	
λ = €15,000	0.762	0.238	
λ = €20,000	0.400	0.600	
λ = €25,000	0.204	0.796	
λ = €30,000	0.119	0.881	
λ = €35,000	0.078	0.922	
λ = €40,000	0.049	0.951	
λ = €45,000	0.033	0.967	

λ or threshold value of the maximum that the health system would be willing to pay per QALY gained. ICC, intra-class coefficient; QALY, quality-adjusted life year.

**Figure 1:**
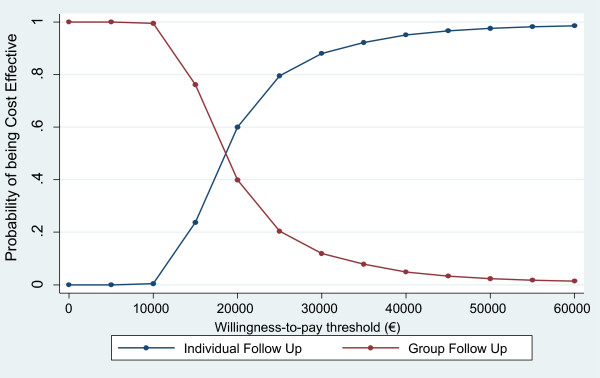
Cost effectiveness acceptability curves for group follow up and individual follow up.

The expected cost effectiveness results are summarised in Table 6 and in Figure 1. At alternative threshold values of €5,000, €15,000, €25,000, €35,000, and €45,000, the probability of group follow-up being cost effective was estimated to be 1.000, 0.762, 0.204, 0.078, and 0.033, respectively.

The results from a series of sensitivity analysis are presented in Additional file 1: Tables S3-S7. The results from these analyses broadly reflect those from the base-case analysis, with group follow-up consistently estimated to be less costly and less effective than individual follow-up.

## Discussion

On the basis of evidence collected alongside a cluster RCT, group follow-up care after the DAFNE programme for type 1 diabetes was, on average, less effective and less costly than standard one-to-one individual follow-up care. These results supplement those from a parallel clinical effectiveness study which reported that group follow-up had no significant effect on the primary outcome of HbA_1c_[4]. Here, we report that group follow-up did not affect quality-adjusted life expectancy at 18 months. Indeed, the QALYs gained outcomes were marginally superior in the individual follow-up arm, although this did not reach statistical significance in the base-case analysis. Taken together, there appears to be no evidence that group follow-up, as trialed in this study, would be a superior strategy post-DAFNE for type 1 diabetes. With respect to costs, group follow-up was associated with a statistically significant saving of €772 per patient, when compared to individual follow-up. Notably, the additional cost of implementing group follow-up was offset by reductions arising from differing patterns of resource use across treatment arms over the course of the trial. This finding suggests that, while group follow-up had no effect on health outcomes, it did have important implications for health service usage and costs of care. Indeed, future studies should explore the cost effectiveness of alternative models of group-based care for this patient cohort.

In terms of the overall cost effectiveness findings, the results for the group follow-up intervention lie in the south west quadrant of the cost effectiveness plane; that is, the intervention is less costly but less effective than the control. In this quadrant, the intervention will be considered cost effective if the amount saved is above the threshold value, λ, per QALY gained [24]. In this case, the results indicate a high probability of the group follow-up intervention being cost effective at low levels of λ but that this declines rapidly as λ increases beyond €10,000. That is, the cost savings associated with group follow-up are significant and are the main driver of cost effectiveness for a range of low threshold values. However, these costs savings are not sufficient to justify cost effectiveness at higher threshold values, where greater weight is placed on improved health outcomes. This is observed graphically in the shape of the CEAC for group follow-up in Figure 1, in that the CEAC cuts the y-axis at a probability of 1.000 and declines sharply as λ increases as the cost savings fall further below the threshold value. The determination of cost effectiveness in this case, therefore, is highly dependent on the threshold value. Unfortunately, the lack of a formal threshold value per QALY gained in Ireland only adds to the uncertainty in the analysis [23]. This notwithstanding, it is unlikely that decision makers in Ireland would consider the adoption of group follow-up given its lack of clinical benefit.

This study adds to the limited literature on the cost effectiveness of complex group-based interventions which seek to improve self-management in patients with type 1 diabetes. A study by Trento and colleagues [25] reported on a 3-year RCT of group care compared to one-to-one clinic visits for individuals with type 1 diabetes. The study randomised 62 patients and delivered 15 group care sessions over 3 years. They reported improvement in knowledge, health behaviours and quality of life and a marginal increase in costs, but no change in HbA_1c_. While a full economic evaluation was not undertaken, the authors concluded that group care was cost effective. Notably, the relatively longer follow-up period in the trial by Trento and colleagues [25] may be an important indicator of why they found improvements in health outcomes and we did not. A second study by Ismail and colleagues [26] reported that group-based motivational enhancement therapy and cognitive behavioural therapy led to an improvement in HbA_1c_ levels at 12 months for individuals with poorly controlled type 1 diabetes. However, the benefits were not observed at 4 years follow-up, thereby indicating that some form of ongoing intervention may be required [27]. Moreover, the economic evaluation at 12 months proved inconclusive [28]. These findings, along with our results, can only add to the outstanding question of how health outcomes for type 1 diabetes, which is a young high-risk population, can be improved.

From an Irish perspective, where the traditional approach to chronic disease management in the community may be described as reactive in nature, our findings highlight the potential resource implications of adopting a more proactive and systematic approach to care. This is particularly relevant given future projections of a growing number of people with chronic disease in Ireland [29], and concerns over the ability of an already resource-constrained healthcare infrastructure to cope with the expected increase in need. Notably, the observed resource usage patterns in this study appear to be true for other chronic conditions including type 2 diabetes and heart disease. For example, a recent Irish RCT evaluated group-based peer support sessions to supplement formal healthcare support for individuals with type 2 diabetes in Irish general practice [30]. While the clinical study showed no significant difference in terms of HbA_1c_, group-based peer support was shown to be cost saving relative to usual care. A second Irish RCT evaluating primary care clinics for the secondary prevention of coronary heart disease in Irish general practice reported significant reductions in hospital admissions and costs of care relative to usual care [31,32]. While further evidence is required, these results suggest a tentative pattern of beneficial resource implications from more proactive approaches to chronic disease management in the community.

There were a number of limitations in this study. Participants were randomised to control and intervention following the collection of baseline data, which indicate that both groups were well matched [4]. However, there was no feasible way to blind the intervention group to participants or to those facilitating the programme and the study is open to a risk of performance bias. Nevertheless, outcome assessment was blinded thus minimising risks to detection bias. As data were collected via structured questionnaires, there are important concerns with respect to participant recall bias at each data collection point. In an attempt to minimise, although admittedly not eradicate, this issue, follow-up data were collected at three consecutive 6-month intervals. With respect to health outcomes, given the lack of Irish utility data for the EQ5D 3 L instrument, the equivalent UK algorithm was adopted and assumed to be generalisable to the Irish population with type 1 diabetes. This may not be the case and reflects the paucity of relevant data for the conduct of economic evaluation in Ireland.

While the cost analysis was conducted from the health service perspective and included a broad range of resource use activities, certain resource items were not captured; for example, the costs of diabetes eye screening were not included. Furthermore, private patient costs such as private health insurance premiums, and broader costs to society such as productivity losses were not captured in the analysis. Nonetheless, there is little to suggest that the inclusion of these resource categories would fundamentally change the results as presented. The process of conducting cost analysis in Ireland is also compromised by the lack of nationally available unit cost data. In estimating unit costs for individual resource activities, we endeavoured at all times to be conservative in any assumptions adopted. It should also be noted that we adopt 2009 prices for the analysis and medical inflation has fallen in the period since the trial was conducted. To examine the uncertainty surrounding the unit cost estimates, sensitivity analysis was conducted in which unit cost data were deflated by 10% and 50%, respectively. Neither set of results fundamentally differed from those of the base-case analysis.

We employed appropriate methods for the statistical analysis of cost and effect data collected alongside cluster RCTs. Notably, clustering for the variables of interest was moderate, with ICCs of 0.016 for total healthcare cost and 0.033 for QALYs gained. Moreover, the correlation coefficient for costs and QALYs gained was −0.18. To account for potential covariate imbalances between treatment arms at baseline [11], we estimated separate linear mixed effects multilevel regression models for costs and QALYs, controlling for a range of covariates and factors. To jointly account for correlation and clustering, we adopted a two-stage non-parametric bootstrapping technique [10]. While the methods adopted were appropriate, arguments could be made for a number of alternative approaches. For example, the adopted linear mixed effects model may be problematic in cases where the distribution of the data deviates significantly from normality. As referenced above, the statistical analysis of cost data is often one such case. That said, the application of more flexible approaches such as generalised estimating equations is problematic when, such as in this study, the number of clusters is small [10,11]. To address concerns over model specification, we conducted a number of sensitivity analyses. The results for the cost analysis estimated using a generalised linear model assuming a Gamma distribution did not differ fundamentally from the base-case analysis. This was also true for the parsimonious analysis, which explored the impact of varying the independent variables in the cost and QALYs regressions models.

As noted above, data were collected via structured questionnaires and, consequently, missing data was an important consideration in the analysis. Details on missing data at each data collection point are presented in Additional file 1: Table S7. After careful consideration of missing data patterns, we proceeded with the assumption that the data were missing at random and multiple imputation was undertaken to impute missing values using the *MI command* in Stata 13. The variables included in the imputation models were pragmatically chosen by the study team and included age, gender, length of illness, treatment arm, and study centre. This approach may be criticised on the basis that values for resource use and EQ5D scores were imputed independently. Furthermore, hospital clinic was included as a fixed effect in the imputation model, reflecting recent guidance that the imputation model should be compatible with the analysis model: that is, both should reflect the multilevel nature of the data [13]. The approach of including the cluster variable as a fixed effect in the imputation model may be problematic in some cases [33]; however, given the small number of clusters in the trial, we deemed it to be appropriate. Indeed, given the moderate ICCs observed, we examined the impact of excluding the cluster effect from the imputation models in sensitivity analyses. Notably, the results were generally consistent with the base-case analysis, although the difference in QALYs gained between group follow-up and individual follow-up became statistically significant. This goes to highlight the importance of applying multilevel imputation models in such cases.

Finally, in the case of chronic disease, a lifetime horizon for analysis is encouraged as interventions may have long-terms implications which occur beyond the end of trial follow-up [6]. Given the absence of improvement in clinical outcomes at 18 months, modelling approaches were not considered in this case and the time horizon of analysis was limited to trial follow-up. Nonetheless, additional follow-up of patients in the trial is essential to explore whether longer term events have a substantive effect on the results presented here.

## Conclusion

There is little evidence to support the implementation of group care as the sole means of follow-up after structured education for individuals with type 1 diabetes. Nonetheless, given the reported cost savings, as well as the growing budget constraints facing health systems worldwide, future studies should continue to explore the clinical and cost effectiveness of alternative models of group care for diabetes.

## Abbreviations

CEAC: cost effectiveness acceptability curve; DAFNE: Dose Adjustment for Normal Eating; HbA_1c_: glycated haemoglobin; ICC: intra-class correlation; QALY: quality-adjusted life year; RCT: randomised controlled trial.

## Competing interests

The authors declare that they have no competing interests.

## Authors’ contributions

SFD, MCOH and EOS conceived the study and participated in the design of the trial and intervention. PG and EOS undertook the acquisition and analysis of the health economic data. All authors participated in critical revision of the manuscript, and have seen and approved the final version.

## Supplementary Material

Additional file 1: Table S1 Comparison of HbA_1c_ between Intervention and Control arms at Baseline and Follow-up. **Table S2.** Completeness of Data at Baseline and Follow Up Time Points. **Table S3.** Sensitivity Analysis 1: Parsimonious Regression Model Results: (1) Incremental Costs estimated controlling for Arm, Baseline Costs and Clustering; (2) Incremental QALYs estimated controlling for Arm, Baseline EQ5D Score and Clustering. **Table S4.** Sensitivity Analysis 2: Alternative Regression Model Specification for the Incremental Cost Analysis: GLM regression model, assuming a GAMMA Variance function, an identity Link Function, and clustered standard errors. **Table S5.** Sensitivity Analysis 3: Assuming that unit costs in Ireland are 10% less than those adopted in the Base-Case Analysis. **Table S6.** Sensitivity Analysis 4: Assuming that unit costs in Ireland are 50% less than those adopted in the Base-Case Analysis. **Table S7.** Alternative Imputation Model Specification Results: Single Level Imputation. Click here for file
