# Supplementary material for: Cost effectiveness of group follow-up after structured education for type 1 diabetes: a cluster randomised controlled trial
Source: Trials. 2014 Jun 14;15:227. doi: 10.1186/1745-6215-15-227 (PMC4070096; doi:10.1186/1745-6215-15-227)
Supplement: Additional file 1: Table S1 — Comparison of HbA1c between Intervention and Control arms at Baseline and Follow-up. Table S2. Completeness of Data at Baseline and Follow Up Time Points. Table S3. Sensitivity Analysis 1: Parsimonious Regression Model Results: (1) Incremental Costs estimated controlling for Arm, Baseline Costs and Clustering; (2) Incremental QALYs estimated controlling for Arm, Baseline EQ5D Score and Clustering. Table S4. Sensitivity Analysis 2: Alternative Regression Model Specification for the Incremental Cost Analysis: GLM regression model, assuming a GAMMA Variance function, an identity Link Function, and clustered standard errors. Table S5. Sensitivity Analysis 3: Assuming that unit costs in Ireland are 10% less than those adopted in the Base-Case Analysis. Table S6. Sensitivity Analysis 4: Assuming that unit costs in Ireland are 50% less than those adopted in the Base-Case Analysis. Table S7. Alternative Imputation Model Specification Results: Single Level Imputation. [file 1745-6215-15-227-S1.doc]

**Supplementary Materials**

**Table 1 - Comparison of HbA_1c_ between Intervention and Control arms at Baseline and Follow-up**

| **Variable/Analysis** | **Baseline** | | **At 18 months Follow-up** | | **Adjusted Treatment Effect**  **(between treatment arms)** | | | **ICC** |
| --- | --- | --- | --- | --- | --- | --- | --- | --- |
| HbA_1c_ (%) | **Mean (SD)** | | **Mean (SD)** | | **Estimate**  **(difference in mean)** | **p-value** | **95% CI** |  |
|  | **Intervention** | **Control** | **Intervention** | **Control** |  |  |  |  |
|  | 8.4 (1.4) | 8.3 (1.3) | 8.4 (1.3) | 8.1 (1.1) | 0.14 | 0.47 | (-0.33, 0.61) | 0.003 |

**Note:** Source- Dinneen et al (4)

**Table 2 – Completeness of Data at Baseline and Follow Up Time Points**

| **Variable/Time Point** | **Baseline: 12 Months**  **N=437** | | **Follow Up 1: 0-6 Months**  **N=437** | | **Follow Up 2: 6-12 Months**  **N=437** | | **Follow Up 3: 12-18 Months**  **N=437** | |
| --- | --- | --- | --- | --- | --- | --- | --- | --- |
|  | **N** | **%** | **N** | **%** | **N** | **%** | **N** | **%** |
| GP Visits: Diabetes  GP Visits: Other  Diabetes Nurse Visits  Diabetes Nurse Calls  Dietician Visits  Dietician Calls  Outpatient Visits: Diabetes  Outpatient Visits: Other  Inpatient Days: Diabetes  Inpatient Days: Other  A&E Visits: Diabetes  A&E Visits: Other  Chiropodist Visits  Diabetes Centre Visits  Quick-Acting Insulin (IUs)  Background Insulin (IUs)  Blood Glucose Tests  Lipid Lowering Therapy  Antiplatelet Therapy  Antihypertensive Therapy | 383  383  382  380  382  382  382  380  383  382  383  382  382  376  436  436  426  428  425  423 | 88  88  87  87  87  87  87  87  88  87  88  87  87  86  99  99  98  98  97  97 | 306  306  307  307  307  304  306  307  307  307  307  306  305  299  298  298  291  292  288  284 | 70  70  70  70  70  70  70  70  70  70  70  70  70  68  68  68  67  67  66  65 | 275  273  272  273  275  275  275  271  274  274  275  275  271  272  302  302  296  296  297  293 | 63  63  62  63  63  63  63  62  63  63  63  63  62  62  69  69  68  68  68  67 | 299  296  298  297  298  297  298  298  299  298  298  299  297  297  289  289  283  283  284  283 | 68  68  68  68  68  68  68  68  68  68  68  68  68  68  66  66  65  65  65  65 |
| EQ5D Score | 428 | 98 | 285 | 65 | 301 | 69 | 289 | 66 |

**Note 1:** IU – Insulin Units

**Table 3 – Sensitivity Analysis 1: Parsimonious Regression Model Results:**

(1) Incremental Costs estimated controlling for Arm, Baseline Costs and Clustering;

(2) Incremental QALYs estimated controlling for Arm, Baseline EQ5D Score and Clustering

| **COST ANALYSIS** | **INTERVENTION** | **CONTROL** | **ICC** |
| --- | --- | --- | --- |
|  | **Group Follow Up**  **N=216** | **Individual Follow Up**  **N=221** |  |
| **Total Healthcare Cost (€)**  Mean (Standard Deviation) | 3548 (405) | 4347 (280) | 0.016 |
|  | **Incremental Analysis**  (Intervention versus Control) | | |
| **Total Healthcare Cost (€)**  Difference in Means (95% CI’s) [p-value**]** | -803 (-1384, -224) [0.007] | | |
|  | | | |
| **EFFECTIVENESS ANALYSIS** | **INTERVENTION** | **CONTROL** | **ICC** |
|  | **Group Follow Up**  **N=216** | **Individual Follow Up**  **N=221** |  |
| **QALYs gained**  Mean (Standard Deviation) | 1.31 (0.12) | 1.35 (0.12) | 0.021 |
|  | **Incremental Analysis**  (Intervention versus Control) | | |
| **QALYs gained**  Difference in Means (95% CI’s) [p-value**]** | -0.03 (-0.08, 0.02) [0.203] | | |
|  | | | |
| **COST EFFECTIVENESS ANALYSIS** | **Probability that Treatment is Cost Effective at λ** | | |
| **Threshold Value (λ)** | **Group Follow Up** | **Individual Follow Up** | |
| **λ = €0** | 1.000 | 0.000 | |
| **λ = €5,000** | 1.000 | 0.000 | |
| **λ = €10,000** | 0.994 | 0.006 | |
| **λ = €15,000** | 0.758 | 0.242 | |
| **λ = €20,000** | 0.479 | 0.521 | |
| **λ = €25,000** | 0.326 | 0.674 | |
| **λ = €30,000** | 0.218 | 0.782 | |
| **λ = €35,000** | 0.167 | 0.833 | |
| **λ = €40,000** | 0.111 | 0.865 | |
| **λ = €45,000** | 0.101 | 0.899 | |

**Table 4 – Sensitivity Analysis 2: Alternative Regression Model Specification for the Incremental Cost Analysis: GLM regression model, assuming a GAMMA Variance function, an identity Link Function, and clustered standard errors.**

| **COST ANALYSIS** | **INTERVENTION** | **CONTROL** | **ICC** |
| --- | --- | --- | --- |
|  | **Group Follow Up**  **N=216** | **Individual Follow Up**  **N=221** |  |
| **Total Healthcare Cost (€)**  Mean (Standard Deviation) | 3543 (718) | 4369 (655) | 0.016 |
|  | **Incremental Analysis**  (Intervention versus Control) | | |
| **Total Healthcare Cost (€)**  Difference in Means (95% CI’s) [p-value**]** | -825 (-1325, -325) [0.002] | | |
|  | | | |
| **EFFECTIVENESS ANALYSIS** | **INTERVENTION** | **CONTROL** | **ICC** |
|  | **Group Follow Up**  **N=216** | **Individual Follow Up**  **N=221** |  |
| **QALYs gained**  Mean (Standard Deviation) | 1.31 (0.12) | 1.35 (0.12) | 0.033 |
|  | **Incremental Analysis**  (Intervention versus Control) | | |
| **QALYs gained**  Difference in Means (95% CI’s) [p-value**]** | -0.04 (-0.08, 0.00) [0.052] | | |
|  | | | |
| **COST EFFECTIVENESS ANALYSIS** | **Probability that Treatment is Cost Effective at λ** | | |
| **Threshold Value (λ)** | **Group Follow Up** | **Individual Follow Up** | |
| **λ = €0** | 1.000 | 0.000 | |
| **λ = €5,000** | 1.000 | 0.000 | |
| **λ = €10,000** | 0.997 | 0.003 | |
| **λ = €15,000** | 0.815 | 0.185 | |
| **λ = €20,000** | 0.456 | 0.544 | |
| **λ = €25,000** | 0.238 | 0.762 | |
| **λ = €30,000** | 0.133 | 0.867 | |
| **λ = €35,000** | 0.087 | 0.913 | |
| **λ = €40,000** | 0.062 | 0.938 | |
| **λ = €45,000** | 0.035 | 0.967 | |

**Table 5– Sensitivity Analysis 3:** Assuming that unit costs in Ireland are 10% less than those adopted in the Base-Case Analysis.

| **COST ANALYSIS** | **INTERVENTION** | **CONTROL** | **ICC** |
| --- | --- | --- | --- |
|  | **Group Follow Up**  **N=216** | **Individual Follow Up**  **N=221** |  |
| **Total Healthcare Cost (€)**  Mean (Standard Deviation) | 3196 (510) | 3903 (496) | 0.016 |
|  | **Incremental Analysis**  (Intervention versus Control) | | |
| **Total Healthcare Cost (€)**  Difference in Means (95% CI’s) [p-value**]** | -695 (-1274, -115) [0.020] | | |
|  | | | |
| **EFFECTIVENESS ANALYSIS** | **INTERVENTION** | **CONTROL** | **ICC** |
|  | **Group Follow Up**  **N=216** | **Individual Follow Up**  **N=221** |  |
| **QALYs gained**  Mean (Standard Deviation) | 1.31 (0.12) | 1.35 (0.12) | 0.021 |
|  | **Incremental Analysis**  (Intervention versus Control) | | |
| **QALYs gained**  Difference in Means (95% CI’s) [p-value**]** | -0.04 (-0.08, 0.00) [0.052] | | |
|  | | | |
| **COST EFFECTIVENESS ANALYSIS** | **Probability that Treatment is Cost Effective at λ** | | |
| **Threshold Value (λ)** | **Group Follow Up** | **Individual Follow Up** | |
| **λ = €0** | 1.000 | 0.000 | |
| **λ = €5,000** | 1.000 | 0.000 | |
| **λ = €10,000** | 0.979 | 0.021 | |
| **λ = €15,000** | 0.617 | 0.383 | |
| **λ = €20,000** | 0.297 | 0.703 | |
| **λ = €25,000** | 0.142 | 0.858 | |
| **λ = €30,000** | 0.084 | 0.916 | |
| **λ = €35,000** | 0.055 | 0.945 | |
| **λ = €40,000** | 0.033 | 0.967 | |
| **λ = €45,000** | 0.023 | 0.977 | |

**Table 6– Sensitivity Analysis 4:** Assuming that unit costs in Ireland are 50% less than those adopted in the Base-Case Analysis.

| **COST ANALYSIS** | **INTERVENTION** | **CONTROL** | **ICC** |
| --- | --- | --- | --- |
|  | **Group Follow Up**  **N=216** | **Individual Follow Up**  **N=221** |  |
| **Total Healthcare Cost (€)**  Mean (Standard Deviation) | 1775 (283) | 2169 (275) | 0.016 |
|  | **Incremental Analysis**  (Intervention versus Control) | | |
| **Total Healthcare Cost (€)**  Difference in Means (95% CI’s) [p-value**]** | -386 (-707, -64) [0.020] | | |
|  | | | |
| **EFFECTIVENESS ANALYSIS** | **INTERVENTION** | **CONTROL** | **ICC** |
|  | **Group Follow Up**  **N=216** | **Individual Follow Up**  **N=221** |  |
| **QALYs gained**  Mean (Standard Deviation) | 1.31 (0.12) | 1.35 (0.12) | 0.021 |
|  | **Incremental Analysis**  (Intervention versus Control) | | |
| **QALYs gained**  Difference in Means (95% CI’s) [p-value**]** | -0.04 (-0.08, 0.00) [0.052] | | |
|  | | | |
| **COST EFFECTIVENESS ANALYSIS** | **Probability that Treatment is Cost Effective at λ** | | |
| **Threshold Value (λ)** | **Group Follow Up** | **Individual Follow Up** | |
| **λ = €0** | 1.000 | 0.000 | |
| **λ = €5,000** | 0.996 | 0.004 | |
| **λ = €10,000** | 0.600 | 0.400 | |
| **λ = €15,000** | 0.119 | 0.881 | |
| **λ = €20,000** | 0.049 | 0.951 | |
| **λ = €25,000** | 0.023 | 0.977 | |
| **λ = €30,000** | 0.014 | 0.986 | |
| **λ = €35,000** | 0.010 | 0.990 | |
| **λ = €40,000** | 0.007 | 0.993 | |
| **λ = €45,000** | 0.004 | 0.996 | |

**Table 7 – Alternative Imputation Model Specification Results: S**ingle Level Imputation.

| **COST ANALYSIS** | **INTERVENTION** | **CONTROL** | **ICC** |
| --- | --- | --- | --- |
|  | **Group Follow Up**  **N=216** | **Individual Follow Up**  **N=221** |  |
| **Total Healthcare Cost (€)**  Mean (Standard Deviation) | 3493 (584) | 4267 (548) | 0.016 |
|  | **Incremental Analysis**  (Intervention versus Control) | | |
| **Total Healthcare Cost (€)**  Difference in Means (95% CI’s) [p-value**]** | -772 (-1352, -191) [0.010] | | |
|  | | | |
| **EFFECTIVENESS ANALYSIS** | **INTERVENTION** | **CONTROL** | **ICC** |
|  | **Group Follow Up**  **N=216** | **Individual Follow Up**  **N=221** |  |
| **QALYs gained**  Mean (Standard Deviation) | 1.31 (0.12) | 1.36 (0.13) | 0.033 |
|  | **Incremental Analysis**  (Intervention versus Control) | | |
| **QALYs gained**  Difference in Means (95% CI’s) [p-value**]** | -0.04 (-0.08, -0.00) [0.035] | | |
|  | | | |
| **COST EFFECTIVENESS ANALYSIS** | **Probability that Treatment is Cost Effective at λ** | | |
| **Threshold Value (λ)** | **Group Follow Up** | **Individual Follow Up** | |
| **λ = €0** | 1.000 | 0.000 | |
| **λ = €5,000** | 1.000 | 0.000 | |
| **λ = €10,000** | 0.982 | 0.018 | |
| **λ = €15,000** | 0.342 | 0.658 | |
| **λ = €20,000** | 0.325 | 0.675 | |
| **λ = €25,000** | 0.134 | 0.866 | |
| **λ = €30,000** | 0.065 | 0.935 | |
| **λ = €35,000** | 0.035 | 0.965 | |
| **λ = €40,000** | 0.022 | 0.978 | |
| **λ = €45,000** | 0.015 | 0.985 | |
